# Friendly foes: The evolution of host protection by a parasite

**DOI:** 10.1002/evl3.19

**Published:** 2017-08-31

**Authors:** Ben Ashby, Kayla C. King

**Affiliations:** ^1^ Department of Mathematical Sciences University of Bath Bath BA2 7AY United Kingdom; ^2^ Department of Integrative Biology University of California Berkeley Berkeley 94720 California; ^3^ Department of Zoology University of Oxford Oxford OX1 3PS United Kingdom

**Keywords:** defensive mutualism, host protection, parasite evolution, parasitism mutualism, resistance, tolerance

## Abstract

Hosts are often infected by multiple parasite species, yet the ecological and evolutionary implications of the interactions between hosts and coinfecting parasites are largely unknown. Most theoretical models of evolution among coinfecting parasites focus on the evolution of virulence, but parasites may also evolve to protect their hosts by reducing susceptibility (i.e., conferring resistance) to other parasites or reducing the virulence of coinfecting parasites (i.e., conferring tolerance). Here, we analyze the eco‐evolutionary dynamics of parasite‐conferred resistance and tolerance using coinfection models. We show that both parasite‐conferred resistance and tolerance can evolve for a wide range of underlying trade‐offs. The shape and strength of the trade‐off qualitatively affects the outcome causing shifts between the minimisation or maximization of protection, intermediate stable strategies, evolutionary branching, and bistability. Furthermore, we find that a protected dimorphism can readily evolve for parasite‐conferred resistance, but find no evidence of evolutionary branching for parasite‐conferred tolerance, in general agreement with previous work on host evolution. These results provide novel insights into the evolution of parasite‐conferred resistance and tolerance, and suggest clues to the underlying trade‐offs in recent experimental work on microbe‐mediated protection. More generally, our results highlight the context dependence of host‐parasite relationships in complex communities.

**Impact Summary**

Hosts are often infected with multiple species of parasites with a variety of evolutionary implications. Do coinfecting parasites evolve to become more or less deadly? Can some parasites evolve to protect their hosts from others, thereby providing a net benefit? Existing theory has largely focused on the first question, but relatively little is known about the evolution of host protection. Empirical evidence indicates that host protection is in fact common; various forms of defense have been observed among fungi, bacteria, protozoa, and viruses (bacteriophages) that colonize hosts. Furthermore, recent experiments have shown that a mildly virulent species of bacteria can evolve to protect animal hosts from a more virulent infection, transitioning along the parasitism‐mutualism continuum. Despite this growing body of empirical research, there are few theoretical predictions for the evolution of host protection. Here, we use mathematical modeling to explore the evolution of two forms of host protection: parasite‐conferred resistance and tolerance. Parasites that confer resistance reduce the likelihood that a second parasite species will be able to infect, whereas parasites that confer tolerance reduce the virulence of coinfecting parasites. We show that both forms of host protection can evolve for a wide range of evolutionary trade‐offs, although there are notable differences between the two and the nature of the trade‐off qualitatively changes the outcome. For example, the generation and maintenance of high and low levels of defense is possible for resistance, but does not appear to be possible for tolerance, consistent with existing theory on host evolution. Our results provide useful insights into the evolution of host protection and make several general predictions (e.g., the coexistence of high and low levels of resistance is more likely when hosts are long‐lived). This study highlights the context‐dependent nature of host–parasite interactions and lays the foundations for future theoretical research on the parasitism–mutualism continuum.

In nature, hosts are typically susceptible to a wide range of parasites, including many species of bacteria and fungi, protozoa, and viruses. Coinfections consisting of multiple strains or species of parasites are therefore likely to be common (Petney & Andrews 1998; Cox 2001; Telfer et al. 2010). Crucially, the dynamics of coinfections can be very different to single infections, both in terms of disease (Griffiths et al. 2011) and evolutionary outcomes (Alizon et al. 2013). For example, infection with *Mycobacterium tuberculosis* (TB) increases the risk of mortality in patients already infected by the human immunodeficiency virus (HIV) (Aaron et al. 2004), but this also decreases the infectious period, which theory predicts may select for increased virulence (Bremermann & Pickering 1983). It is clear that understanding how coinfecting parasites interact with each other and their hosts has important implications not only for infectious disease control (Brown et al. 2009; Balmer & Tanner 2011; Griffiths et al. 2011), but also for understanding the ecological and evolutionary outcomes of the community (Read & Taylor 2001; Brown et al. 2002; Alizon 2013; Johnson et al. 2015).

The literature on coinfections has predominantly focused on the evolution of virulence (reviewed in Alizon et al. 2013). In general, theory predicts that low (high) relatedness during coinfections selects for higher (lower) virulence (Hamilton 1972; Bremermann & Pickering 1983; Sasaki & Iwasa 1991; Frank 1992, 1994, 1996; van Baalen & Sabelis 1995). The core assumption of these models is that parasites interact indirectly through exploitative competition (one parasite indirectly harms the prospects of another by consuming a shared resource), but parasites can interact through many other mechanisms. For example, phenotypic plasticity and impaired host immunity select for lower virulence (Choisy & de Roode 2010), and if cooperation among kin increases growth rates then high relatedness may increase virulence (Chao et al. 2000; Brown et al. 2002; West & Buckling 2003). Alternatively, parasites may modulate the virulence of coinfecting species to prolong the life of the host, or may secrete antimicrobial toxins that actively harm competitors through interference competition (spite). For instance, *Streptococcus pneumoniae* produces hydrogen peroxide, which induces lysogenic bacteriophage in *Staphylococcus aureus* to lyse their hosts (Selva et al. 2009). Interference competition has received much less attention than exploitative competition, but is predicted to play a crucial role in parasite evolution (Gardner et al. 2004). For example, spite selects for greater virulence when relatedness is at an extreme and lower virulence when relatedness is intermediate (Gardner et al. 2004; Massey et al. 2004; Inglis et al. 2009). The ability of parasites to protect their host from additional, perhaps more virulent, infections may therefore evolve as a by‐product of interference competition.

Host protection has been found across plant and animal species (Ford & King 2016). Although protective microbes can also be parasitic and therefore costly, they may provide a net benefit to their hosts if they compete with more virulent parasites—“the enemy of my enemy is my friend” (Martinez et al. 2015). Protective microbes can form a significant component of host defense. For example, the survival of monarch butterfly larvae (*Danaus plexippus*) is higher when coinfected with a virulent protozoan parasite (*Ophryocystis elektroscirrha*) and a lethal parasitoid fly (*Lespesia archippivora*), than when only infected by the latter (Sternberg et al. 2011). Some vertically transmitted bacteria in insects, such as *Hamiltonella* (Vorburger & Gouskov 2011; Polin et al. 2014) and *Wolbachia* (Hughes et al. 2011; Blagrove et al. 2012), are costly but provide hosts with protection against other parasite species. Other known examples of parasite‐conferred defense include the transfer of resistance genes by lysogenic phages (van Baalen & Jansen 2001) and protection against a virulent fungus by less virulent fungi (Michalakis et al. 1992). Recently, it was discovered that within‐host antagonistic interactions between microbial parasite species drove the rapid de novo evolution of protective properties in a worm–bacteria system (King et al. 2016). The boundary between parasitism and mutualism is often blurred, with many bacteria providing context‐dependent defense and retaining mild pathogenicity (Polin et al. 2014; Martinez et al. 2015). Together, these empirical observations suggest that evolutionary transitions between parasitism and mutualism are likely to be common. Moreover, this work highlights the potential for host protection to impact infectious disease ecology and evolution.

Few theoretical predictions exist to support this growing body of empirical research on the evolution of host protection (Michalakis et al. 1992; van Baalen & Jansen 2001; Jones et al. 2011). Here, we show that host protection can readily evolve, but the precise outcome depends on the shape and strength of any underlying trade‐offs.

## Methods

We study the evolution of host protection using two coinfection models (Choisy & de Roode 2010; Alizon 2013). First, we assume that coinfections only occur between parasites of different species (model A), as this greatly simplifies the analysis. Hence if a mutant strain arises in a given host, we assume that it is either immediately cleared or replaces the resident strain. We relax this assumption in the Supporting Information (model B), allowing coinfections to occur between strains of the same species.

### MODEL DESCRIPTION

In our primary model (model A), the host population is divided into four classes according to its infection status: susceptible to both parasite species (*S*); infected by parasite 1 but susceptible to parasite 2 (*I*_1_); infected by parasite 2 but susceptible to parasite 1 (*I*_2_); and infected by both parasites (*I*_12_). Hosts have a natural mortality rate of *b* and reproduce at a maximum per‐capita rate of *a* subject to density‐dependent competition (qN with $N=S+I_{1}+I_{2}+I_{12}$) giving a birth rate of ν(N)=(a−qN)N. The maximum pairwise transmission rate for parasite *j* is ${\tilde{\beta }}_{j}$ and recovery occurs at rate $\gamma_{j}$; there is no immunity following recovery. Hosts experiencing a single infection by parasite *j* suffer an additional baseline mortality rate (virulence) of ${\tilde{\alpha }}_{j}$, while coinfections lead to an additional mortality rate of α_12_.

We study the evolution of two forms of host protection by parasite 1: (i) resistance, $\beta_{2}\left(y\right)={\tilde{\beta }}_{2}\left(1-\delta y\right)$; and (ii) tolerance, $\alpha_{12}\left(y\right)=\alpha_{1}\left(y\right)+{\tilde{\alpha }}_{2}\left[1-(1-\delta )y\right]$, with δ=0 or δ=1. The strength of host protection is denoted by 0≤y≤1, with y=0 corresponding to no protection and y=1 to maximum protection. Hence, infection by parasite 1 may either reduce susceptibility to subsequent infection by parasite 2 (resistance, δ=1), or reduce the virulence of parasite 2 in mixed infections (tolerance, δ=0). For example, parasite 2 may struggle to establish itself in hosts that are already infected by parasite 1, or parasite 1 may actively harm parasite 2 through physiological defenses (resistance). Alternatively, parasite 1 may produce antitoxins that limit virulence factors produced by parasite 2 (tolerance). Parasites that protect their hosts incur a fitness cost, c(y), which leads to either a reduction in transmission, $\beta_{1}\left(y\right)={\tilde{\beta }}_{1}\left[1-c(y)\right]$, or an increase in virulence, $\alpha_{1}\left(y\right)={\tilde{\alpha }}_{1}\left[1+c(y)\right]$, where

(1) $$c\left(y\right)=\frac{c_{1}\left(1-e^{c_{2}y}\right)}{1-e^{c_{2}}}$$

The parameter $c_{1}>0$ determines the maximum strength of the cost and $c_{2}\in R_{\neq 0}$ determines the rate at which costs increase (accelerating: $c_{2}>0$, decelerating: $c_{2}<0$). Costs associated with host protection may arise due to changes in either the allocation or consumption of host resources. For example, the protective parasite may divert resources from making transmission stages to producing antimicrobials or antivirulence compounds (transmission cost). Alternatively, a parasite may cause additional damage to the host by consuming more resources so that it can maintain its transmission rate and defend against another parasite (virulence cost). It is possible that both transmission and virulence will vary with host protection, but the results are likely to be similar to the single‐cost scenarios (e.g., if virulence increases/decreases in addition to a transmission rate cost then the overall cost is slightly stronger/weaker compared to when virulence is fixed). We therefore only consider single costs.

The epidemiological dynamics of monomorphic parasites in well‐mixed populations are:

(2a) $$\frac{\mathrm{dS}}{\mathrm{dt}}=\nu \left(N\right)-\left[b+\lambda_{1}\left(y\right)+\lambda_{2,S}\right]S+\gamma_{1}I_{1}+\gamma_{2}I_{2}$$

(2b) $$\frac{{\mathrm{dI}}_{1}}{\mathrm{dt}}=\lambda_{1}\left(y\right)S-\left[\Gamma_{1}\left(y\right)+\lambda_{2}\left(y\right)\right]I_{1}+\gamma_{2}I_{12}$$

(2c) $$\frac{{\mathrm{dI}}_{2}}{\mathrm{dt}}=\lambda_{2,S}S-\left[\Gamma_{2}+\lambda_{1}\left(y\right)\right]I_{2}+\gamma_{1}I_{12}$$

(2d) $$\frac{{\mathrm{dI}}_{12}}{\mathrm{dt}}=\lambda_{1}\left(y\right)I_{2}+\lambda_{2}\left(y\right)I_{1}-\Gamma_{12}\left(y\right)I_{12}$$

where $\Gamma_{1}\left(y\right)=b+\alpha_{1}\left(y\right)+\gamma_{1}$, $\Gamma_{2}=b+{\tilde{\alpha }}_{2}+\gamma_{2}$, and $\Gamma_{12}\left(y\right)=b+\alpha_{12}\left(y\right)+\gamma_{1}+\gamma_{2}$ are the inverse of the infectious periods, and $\lambda_{2,S}={\tilde{\beta }}_{2}\left(I_{2}+I_{12}\right)$ and $\lambda_{j}\left(y\right)=\beta_{j}\left(y\right)\left(I_{j}+I_{12}\right)$ are the forces of infection (j=1,2). The initial dynamics of a rare mutant, $y_{m}$, when the resident is at equilibrium $\left(N^{*}=S^{*}+I_{1}^{*}+I_{2}^{*}+I_{12}^{*}\right)$ are:

(3a) $$\frac{{\mathrm{dI}}_{m}}{\mathrm{dt}}=\lambda_{1}\left(y_{m}\right)S^{*}-\left[\Gamma_{1}\left(y_{m}\right)+\lambda_{2}^{*}\left(y_{m}\right)\right]I_{m}+\gamma_{2}I_{m2}$$

(3b) $$\frac{{\mathrm{dI}}_{m2}}{\mathrm{dt}}=\lambda_{1}\left(y_{m}\right)I_{2}^{*}+\lambda_{2}^{*}\left(y_{m}\right)I_{m}-\Gamma_{12}\left(y_{m}\right)I_{m2}$$

where $I_{m}$ is hosts infected with the mutant and $I_{m2}$ is hosts coinfected with the mutant and parasite 2.

### ANALYSIS

We use a combination of numerical analysis and simulations to explore the evolution of host protection. Using evolutionary invasion analysis (Metz et al. 1992; Dieckmann & Law 1996; Geritz et al. 1998), we first derive the fitness of a rare mutant, $w(y_{m})$—assumed to be phenotypically similar to the resident for parasite 1—when the resident population is at equilibrium. Since there is no analytic solution for the multiparasite endemic equilibrium, we solve the system of equations over a sufficiently long time period to ensure that the system is close to a stable state (verified numerically). The population will evolve in the direction of the selection gradient, $s\left(y\right)=\frac{dw}{dy_{m}}{|}_{y_{m}=y}$, until a singular strategy, $y^{*}$, is reached at $s(y^{*})=0$. The singular strategy is locally "evolutionarily stable" (ES) if $\frac{ds}{dy}{|}_{y=y^{*}}<0$ and is "convergence stable" (CS) if s(y)<0 for $y=y^{*}+\varepsilon$ and s(y)>0 for $y=y^{*}-\varepsilon$ for sufficiently small ε>0. ES implies that a singular strategy is a local fitness maximum and CS implies that the strategy is locally attracting (i.e., it can be reached by recurrent small mutations). We evaluate whether $y^{*}$ is ES and CS, in which case it is a "continuously stable strategy" (CSS). If $y^{*}$ is CS but not ES, then the singular strategy is a branching point (BR), which indicates that disruptive selection will occur leading to a protected dimorphism. If $y^{*}$ is neither CS nor ES, then the singular strategy is a repeller (RE), which may lead to bistability (i.e., the outcome depends on the initial conditions). If a repeller is the only singular strategy, then y=0 and y=1 are both locally attracting. Global minimisation (MN) occurs when s(y)<0 for all y>0, and global maximization (MX) occurs when s(y)>0 for all y<1. Finally, the singular strategy is referred to as a "Garden of Eden" when $y^{*}$ is ES but is not CS (the singular strategy is evolutionarily stable but is unattainable through small mutations).

The above method assumes a separation of ecological and evolutionary timescales (mutations are rare) and that selection is weak (mutations have a small effect). We relax these assumptions in our simulations, which allow mutations to occur when the system is not close to its dynamical attractor (simulation code in the online Supporting Information). Starting with a single resident trait, $y_{r}$, we solve the ODE system for a given time period [0, *T*] (T=100), then introduce a mutant, $y_{m}=y_{r}\pm \varepsilon_{1}$ (mutation size $\varepsilon_{1}=0.02$), at low frequency. We then rerun the ODE solver over the period [T,2T] and remove any strains that have fallen below a frequency of $\varepsilon_{2}=10^{-3}$. If more than one trait is still present in the population, then the next mutant is chosen based on a weighted probability of the trait frequencies. The process is repeated for n=2000 iterations.

## Results

### IMPACT OF HOST PROTECTION ON THE ECOLOGICAL DYNAMICS

We begin by examining how host protection affects the ecological dynamics by analyzing the basic reproductive ratios, $R_{0}\left(i,j\right)$, which give the average number of secondary infections for parasite *j* when rare given that parasite *i* is already at equilibrium (Choisy & de Roode 2010). The equations for $R_{0}\left(i,j\right)$ are (see Supporting Information):

(4a) $$\begin{array}{c} R_{0}\left(2,1\right)=\frac{\beta_{1}\left(y\right)\left(\beta_{2}\left(y\right)I_{2}^{*}\left(S^{*}+I_{2}^{*}\right)+I_{2}^{*}\left(\Gamma_{1}\left(y\right)+\gamma_{2}\right)+\Gamma_{12}\left(y\right)S^{*}\right)}{\beta_{2}\left(y\right)I_{2}^{*}\left(\Gamma_{12}\left(y\right)-\gamma_{2}\right)+\Gamma_{1}\left(y\right)\Gamma_{12}\left(y\right)} \end{array}$$

(4b) $$\begin{array}{c} R_{0}\left(1,2\right)=\frac{{\tilde{\beta }}_{2}S^{*}\left(\beta_{1}\left(y\right)I_{1}^{*}+1\right)+\beta_{2}\left(y\right)I_{1}^{*}\left(\beta_{1}\left(y\right)I_{1}^{*}+\Gamma_{2}+\gamma_{1}\right)}{\Gamma_{12}\left(y\right)\left(\beta_{1}\left(y\right)I_{1}^{*}\left(\Gamma_{12}\left(y\right)-\gamma_{1}\right)+\Gamma_{2}\Gamma_{12}\left(y\right)\right)} \end{array}$$

**Figure 1:**
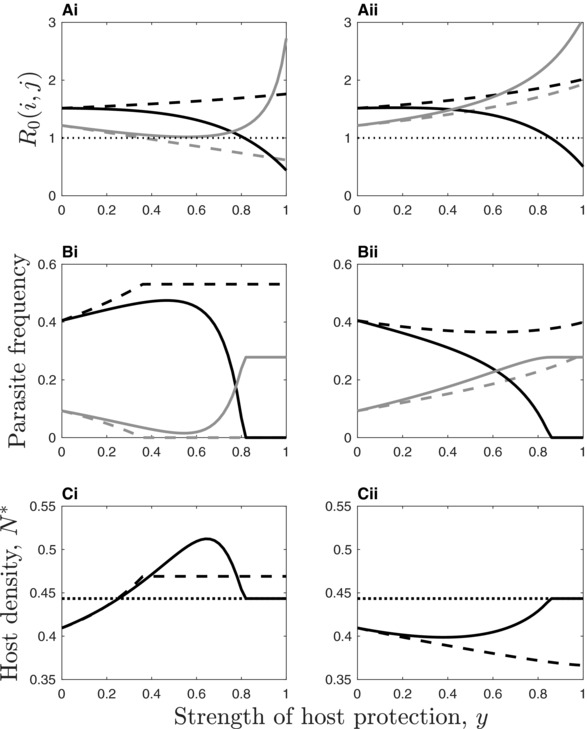
Impact of parasite‐conferred resistance (left) and tolerance (right) on the ecological dynamics. Dashed lines correspond to $c_{1}=0$ (no costs) and solid lines to accelerating transmission rate costs $\left(c_{1}=0.75,c_{2}=3\right)$. (Ai–ii) The basic reproductive ratio, $R_{0}\left(i,j\right)$, of parasite j=1,2 (black and gray, respectively) when parasite i=2,1 is at equilibrium (the dotted line shows the exclusion threshold). (Bi–ii) Parasite frequency at equilibrium. (Ci–ii) Host density at equilibrium (the protective parasite is a net mutualist when host density is above the dotted line). Parameters: a=1, b=0.5, q=0.5, ${\tilde{\alpha }}_{1}=0.5$, ${\tilde{\alpha }}_{2}=1$, ${\tilde{\beta }}_{1}=5$, ${\tilde{\beta }}_{2}=5$, $\gamma_{1}=0.1$, $\gamma_{2}=0.1$.

When the other parasite is not present equations 4A–B reduce to $R_{0}\left(1\right)=\frac{\beta_{1}\left(y\right)S^{*}}{\Gamma_{1}\left(y\right)}$ and $R_{0}\left(2\right)=\frac{{\tilde{\beta }}_{2}S^{*}}{\Gamma_{2}}$, respectively. The parasites coexist at a stable endemic equilibrium provided both $R_{0}\left(i,j\right)>1$, but if $R_{0}\left(i,j\right)<1$ for one parasite then it will be excluded. In general, tolerance increases *R*_0_(1, 2) and the prevalence of parasite 2 (Fig. 1A and B), as is the case with single parasite systems (Boots et al. 2009). From the perspective of the host, the benefits of parasite‐conferred tolerance are likely to be rather limited, as increased survival at the individual level leads to increased disease prevalence at the population level; the net effect may therefore be negative for the host (Fig. 1C). For parasite‐conferred resistance, both *R*_0_(1, 2) and the prevalence of parasite 2 initially decline as host protection increases, but if host protection is costly then the prevalence of parasite 1 will eventually fall, causing a resurgence for parasite 2 (Fig. 1A and B). This means that stronger resistance can increase the prevalence of parasite 2, although such a situation is unlikely to be evolutionarily stable. Parasite‐conferred resistance can be extremely beneficial for the host, leading to a marked increase in host density at equilibrium (Fig. 1C).

### PARASITE FITNESS AND SELECTION GRADIENT

Using the next‐generation method (Hurford et al. 2010), we derive the following expression which is sign equivalent to the invasion fitness of a rare mutant, $y_{m}$ (see Supporting Information):

(5) $$w\left(y_{m}\right)=\frac{\beta_{1}\left(y_{m}\right)A\left(y_{m}\right)}{B\left(y_{m}\right)}-1$$

where A(ym)=S∗[Γ12(ym)+λ2∗(ym)]+I2∗[Γ1(ym)+γ2+λ2∗(ym)] and B(ym)=Γ12(ym)[Γ1(ym)+λ2∗(ym)]−γ2λ2∗(ym). The selection gradient, $s\left(y\right)=\frac{dw}{dy_{m}}{|}_{y_{m}=y}$, is:

(6) $$\begin{array}{ccc} s\left(y\right) & = & \frac{1}{B\left(y\right)}\left\{A\left(y\right){\left.\frac{d\beta_{1}}{dy_{m}}\right|}_{y_{m}=y}+\beta_{1}\left(y\right){\left.\frac{dA}{dy_{m}}\right|}_{y_{m}=y}\right.\\ & & \left.-\frac{\beta_{1}\left(y\right)A\left(y\right)}{B\left(y\right)}{\left.\frac{dB}{dy_{m}}\right|}_{y_{m}=y}\right\} \end{array}$$

We solve the selection gradient and its derivative numerically to determine whether each singular strategy is ES and/or CS. We primarily consider the effects of the strength and shape of the trade‐off (eq. (1)), along with the effects of host lifespan (1/b) and the virulence of parasite 1 $\left({\tilde{\alpha }}_{1}\right)$. We focus on transmission rate costs in the main text and virulence costs in the Supporting Information. The Supporting Information also contains the results for model B, which are broadly consistent with those presented here.

### EVOLUTION OF PARASITE‐CONFERRED RESISTANCE

**Figure 2:**
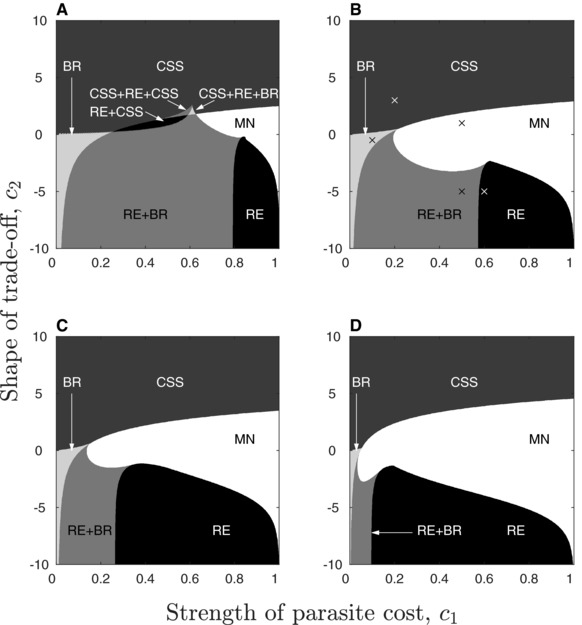
Evolution of parasite‐conferred resistance when there is a transmission rate cost. Higher values of *c*_1_ correspond to greater costs, and higher (lower) values of *c*_2_ correspond to more strongly accelerating (decelerating) costs (eq. (1)). Qualitative outcomes: minimisation (MN); intermediate continuously stable strategy (CSS); repeller (RE); and evolutionary branching (BR). The natural mortality rate, *b*, increases from 0.05 (left column) to 0.5 (right column). The virulence of parasite 1, ${\tilde{\alpha }}_{1}$, increases from 0.1 (top row) to 1 (bottom row). Crosses in panel B correspond to Fig. 3. Remaining parameters as described in Fig. 1.

**Figure 3:**
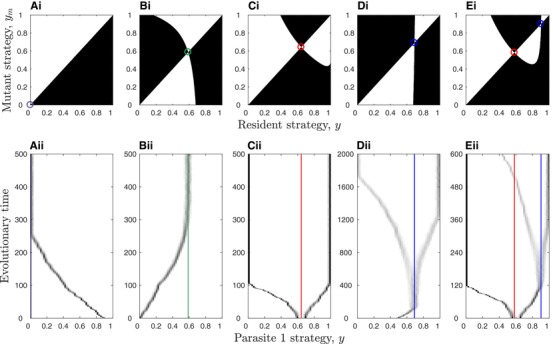
Pairwise invasion plots (PIPs; top) and simulations (bottom) for the points in Fig. 2B: (A) minimisation (purple); (B) CSS (green); (C) repeller (red); (D) evolutionary branching (blue); (E) repeller (red) and evolutionary branching (blue). The mutant can only invade in the black regions of the PIPs, which means that *y* increases (decreases) when the region immediately above (below) the line $y=y_{m}$ is black and the region immediately below (above) this line is white. Note that plots C and E show two separate simulations with different initial conditions either side of the repeller. Same parameters as Fig. 1, with ${\tilde{\alpha }}_{1}=0.1$.

Assuming parasite 1 initially confers no protection to the host, resistance can only evolve by small mutations when the trade‐off accelerates $\left(c_{2}>0\right)$, or when the trade‐off decelerates and the cost is small $(c_{1}\ll 1,c_{2}<0).$ The qualitative outcome is most sensitive to the shape of the trade‐off (*c*_2_), and there are five regions of the trade‐off space that are common (Figs. 2 and 3). First, the parasite may always experience selection against host protection (minimisation). This occurs for moderate to high costs over a fairly broad range of intermediate trade‐off shapes. Second, the parasite may evolve to an intermediate level of host protection (CSS) when costs accelerate $\left(c_{2}>0\right)$. Third, a repeller may cause bistablility so that the parasite evolves to either minimize or maximize host protection depending on the initial conditions (y=0 and y=1 are locally attracting). This outcome generally occurs when costs decelerate $\left(c_{2}<0\right)$ and are relatively large $\left(c_{1}\gg 0\right)$. Fourth, the parasite may branch into two strategies through disruptive selection, eventually leading to a stable dimorphism with $y_{1}^{*}=0$ and $y_{2}^{*}=1$. Branching occurs when costs decelerate and are relatively low in magnitude $\left(c_{1}\ll 1\right)$. Finally, there may be two singular strategies: a repeller and a branching point. In all cases we found that the repeller was located below the branching point. Hence, the parasite may either minimize *y* (y=0 is a local attractor) or branch into two diverging strategies depending on the initial conditions. This outcome occurs for intermediate decelerating costs.

Increasing the lifespan of the host (decreasing *b*) and reducing the virulence of parasite 1 (decreasing ${\tilde{\alpha }}_{1}$) generally increases the size of the branching regions and makes minimisation and bistability less likely. However, for sufficiently low *b* and ${\tilde{\alpha }}_{1}$ we found more complex outcomes for intermediate costs that are weakly accelerating, consisting of a repeller and either one or two CSSs, or a CSS and a branching point (Fig. 2A). These regions are mostly similar to the RE and RE + BR regions described above, with the exception that y=0 and y=1 are no longer local attractors. We verified the numerical analysis of the model with simulations and found them to closely match the numerical results (Fig. 3).

### EVOLUTION OF PARASITE‐CONFERRED TOLERANCE

**Figure 4:**
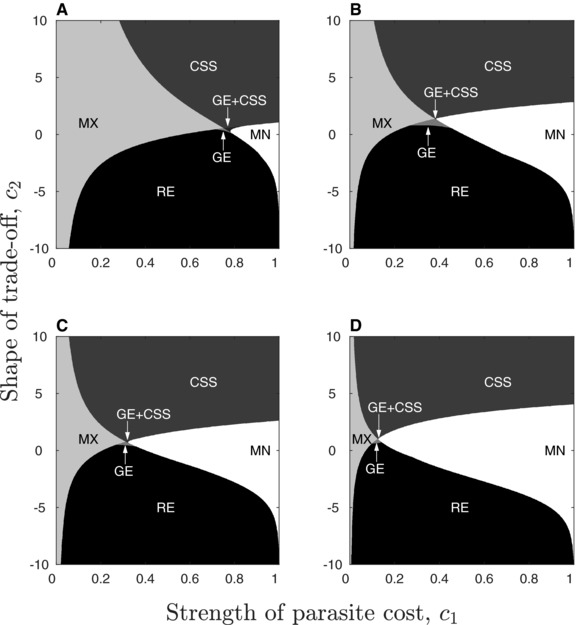
Evolution of parasite‐conferred tolerance when host protection is associated with a transmission rate cost. In addition to most of the singular strategies described in Fig. 2 for parasite‐conferred resistance, we also find: maximization (MX) for nonzero costs and Garden of Eden (GE) with or without a CSS. The natural mortality rate, *b*, increases from 0.05 in the plots on the left to 0.5 on the right. The virulence of parasite 1, ${\tilde{\alpha }}_{1}$, increases from 0.1 in the top row to 1 in the bottom row. The cost function and the remaining labels and parameters are described in Fig. 2.

**Figure 5:**
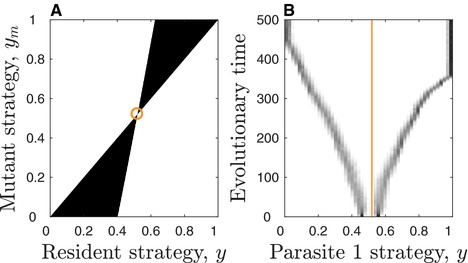
PIP (A) and simulation (B) for the Garden of Eden (GE; orange) outcome. The GE is evolutionarily stable but is not convergence stable, and hence it cannot be approached by small mutations (the mutant can only invade the resident in the black regions of the PIP). In reality, the GE will generally behave as a repeller, as shown in the two evolutionary trajectories in panel B. Parameters as described in Fig. 1, except $c_{1}=0.37$, $c_{2}=1$, ${\tilde{\alpha }}_{1}=0.1$, and δ=0.

As with resistance, the qualitative outcome for tolerance is most sensitive to the shape of the trade‐off (Fig. 4), and tolerance can only evolve by small mutations when the trade‐off accelerates $\left(c_{2}>0\right)$, or when the trade‐off decelerates and the cost of protection is small $\left(c_{1}\ll 1,c_{2}<0\right)$. However, there are some notable differences between the two scenarios. When parasite 1 confers tolerance there are four main regions of the cost space describing different evolutionary outcomes (Fig. 4). First, the parasite always experiences selection against host protection (minimisation) when costs are moderate to high (over a broad range of intermediate trade‐off shapes). Second, selection always favors greater host protection (maximization) when costs are low to moderate in magnitude, regardless of whether the trade‐off accelerates or decelerates. Third, the parasite may evolve an intermediate level of host protection (CSS) for moderate to high accelerating costs. Fourth, the system may exhibit bistability due to a repeller. Bistability usually occurs for intermediate decelerating costs, although the region of bistability shrinks as the shape of the trade‐off tends toward being linear $\left(c_{2}\to 0\right)$. A small region of the cost space exists near the intersection of these main regions corresponding to a Garden of Eden scenario (with or without a CSS). This means that the singular strategy is evolutionarily stable but is unattainable through small mutations, and so in reality it is likely to behave as a repeller (Fig. 5).

These general relationships are consistent as host lifespan and the virulence of parasite 1 are varied, although maximization tends to become more likely as *b* and ${\tilde{\alpha }}_{1}$ decrease. We did not find any evidence of evolutionary branching when the parasite confers tolerance, which by contrast is relatively common in the case of resistance. Again, simulations were found to closely match the numerical results (similar to Fig. 3, omitted for brevity).

## Discussion

Interactions between coinfecting parasites are likely to play a crucial role in shaping the ecological and evolutionary dynamics of infectious diseases (Read & Taylor 2001; Brown et al. 2002; Alizon 2013; Johnson et al. 2015). A large body of theory has primarily focused on how competitive or cooperative strategies to exploit host resources affect the evolution of virulence in mixed infections (Bremermann & Pickering 1983; Sasaki & Iwasa 1991; Frank 1992, 1996; van Baalen & Sabelis 1995; Alizon & van Baalen 2008; Choisy & de Roode 2010; Alizon & Lion 2011). The aim of our study was to understand the extent to which parasite inter‐ and intraspecies interactions drive the evolution of host protection, a widely observed phenomenon (Michalakis et al. 1992; van Baalen & Jansen 2001; Ford & King 2016). Our study was therefore more closely related to theoretical models of spite (Gardner et al. 2004) and an existing model of host protection by vertically transmitted parasites (Jones et al. 2011).

We explored how host protection evolves subject to a wide range of trade‐offs. Our study has two key results. First, host protection can evolve for many types of trade‐off, but the qualitative outcome depends on the mechanism of protection and the precise nature of the trade‐off. For example, evolutionary branching—leading to a stable dimorphism—only appears to occur for parasite‐conferred resistance, not for tolerance. This is likely due to the positive frequency dependence that is typically associated with tolerance mechanisms, which leads to an increase in the prevalence of the targeted parasite and tends to prevent branching (Roy & Kirchner 2000; Boots et al. 2009). In general, host protection is most likely to evolve if the trade‐off accelerates, or if the trade‐off decelerates and the cost of protection is relatively low. The qualitative outcome is more sensitive to the shape of the trade‐off rather than the magnitude of the cost, with accelerating trade‐offs generally selecting for a CSS, whereas decelerating trade‐offs tend to produce evolutionarily unstable strategies, leading to either bistability or branching. These patterns are consistent with general theory in adaptive dynamics, which shows that strongly accelerating trade‐offs produce CSSs and strongly decelerating trade‐offs produce evolutionary repellers (Mazancourt & Dieckmann 2004; Bowers et al. 2005).

Our second key result is that longer host lifespans and lower virulence of the protective parasite tend to increase the range of conditions that lead to evolutionary branching (resistance) or maximization (tolerance). In both cases, there is an increase in the average infectious period and hence in the likelihood of coinfections. It is easy to see that reducing the background mortality (*b*) or virulence from parasite 1 $\left({\tilde{\alpha }}_{1}\right)$ will select for greater tolerance because the virulence of the second parasite then dominates the infectious period for coinfections. It is less clear why reducing these parameters increases the likelihood of evolutionary branching, but this pattern is consistent with a previous study of host–parasite range coevolution which showed that branching is more common as host lifespan (and hence the infectious period) increases (Best et al. 2010). Together, our results predict that host protection can readily evolve under a wide variety of circumstances. Moreover, the broad patterns we observe in our model are consistent with previous theory on the evolution of resistance and tolerance by the host (Boots & Bowers 1999; Boots et al. 2009). The key difference here, however, is that defense is conferred by the parasite, and thus is obtained from the environment dynamically rather than being genetically inherited. Such situations are likely to be common in natural populations, which typically consist of complex communities of parasites that may confer context‐dependent costs and benefits to their hosts (Michalakis et al. 1992; van Baalen & Jansen 2001; Betts et al. 2016; Ford & King 2016).

Our study builds on previous models of coinfecting parasites, in particular the work of Choisy and de Roode (2010) (model A) and Alizon (2013) (model B). A crucial difference between the two models is that different strains of the same species are able to coinfect the same host in model B. Still, we found that our results were remarkably similar across the two frameworks (Fig. S1). In model B, we assumed that defense is specific to parasite 2, but if defense is more general (e.g., a priority effect), then other strains of parasite 1 are also likely to be negatively impacted. We also assumed that the overall level of resistance or tolerance was equal to the mean of the two coinfecting strains of parasite 1, but it is possible that the results may differ for other functional forms. A more realistic (but much more complex) approach would be to use a nested model of within‐ and between‐host dynamics to fully account for the dynamics of coinfecting strains (Mideo et al. 2008). Future theory should examine whether the evolution of parasite‐conferred resistance and tolerance is affected by within‐host dynamics.

The biological arguments underlying our results are fairly intuitive. Parasites should not only evolve optimal strategies to exploit host resources, but should also evolve strategies to cope with mixed infections. While many studies of parasite evolution have considered coinfections, the motivation of our study is different to most of the preceding work, which has focused almost exclusively on the evolution of virulence. In our model, virulence does not evolve, and thus it is not the degree of host exploitation that is under selection. Rather, it is the degree to which the focal parasite defends a common resource and the mechanism by which the resource is protected that is evolvable. In spite of this key difference, there are some conceptual similarities with the evolution of virulence theory. In particular, the mechanism by which the coinfecting parasites interact with each other and the host is crucial (Bremermann & Pickering 1983; van Baalen & Sabelis 1995; Frank 1996; West & Buckling 2003; Gardner et al. 2004; Choisy & de Roode 2010). Here, host protection is likely to have a negative impact on the nonprotective parasite if the mechanism in question leads to interference competition (e.g., resistance), but conversely may be beneficial if host protection extends the longevity of mixed infections (e.g., tolerance). Interestingly, most documented examples of host protection involve interference competition as the mechanism at play (Ford & King 2016). The context of the interaction between coinfecting parasites is clearly crucial for predicting the ecological and evolutionary outcomes in both cases. Our study is also related to recent work on the impact of superinfections on host evolution (Kada & Lion 2015; Donnelly et al. 2017). Again, a common theme is that the nature of the interaction between parasites and their relative virulence can have important consequences for the evolution of defense, regardless of whether this is intrinsic to the host or conferred by another species.

We are only aware of one other theoretical model of the evolution of host protection by another species, which concerned the resistance conferred by vertically transmitted symbionts against horizontally transmitted parasites (Jones et al. 2011). The studies are clearly linked by the common theme of host protection, although there are notable differences (e.g., in our model defense may take the form of either resistance or tolerance and the protective parasite is transmitted horizontally). In particular, Jones et al. (2011) considered the impact of parasitic castration on the level of host defense, which is crucial because the defensive parasite is transmitted vertically, and hence its reproduction is intrinsically linked to that of the host. The impact of parasitic castration is likely to be much lower in our model, as both parasites are transmitted horizontally.

Our study is closely linked to recent empirical work showing the de novo evolution of microbe‐mediated protection during experimental evolution of a novel, tripartite interaction between a host, and two parasites (King et al. 2016). This work showed that mildly parasitic bacteria (*Enterococcus faecalis*) living in nematodes rapidly evolve to defend their animal hosts against infection by a more virulent pathogen (*S. aureus*). Driven by frequent antagonistic interactions with coinfecting *S. aureus*, *E. faecalis* evolve to increase production of superoxides. These act as antimicrobials, which actively suppress the virulence and within‐host fitness of *S. aureus*. The evolved microbes also stay mildly parasitic during single infections, demonstrating the context‐dependent nature of their beneficial effects.

The theory established in the present study adds to our general understanding of the complex ecoevolutionary relationships between hosts and coinfecting parasites, and specifically, to our understanding of evolution along the mutualism–parasitism continuum (Michalakis et al. 1992; van Baalen & Jansen 2001). For simplicity, we considered the evolution of either parasite‐conferred resistance (δ=1) or tolerance (δ=0). However, some parasites may confer mixed modes of protection to their hosts (0<δ<1), in which case it is likely that the level of investment in each mode of defense may evolve. An interesting extension of our work would therefore be to allow both the strength and level of investment in each mode of host protection to coevolve. We have addressed the question of how different mechanisms of host protection evolve when hosts and nonprotective parasites are evolutionarily static, but such a constraint will need to be relaxed in future theory to understand the coevolutionary dynamics of all parties. For example, selection for mechanisms that reduce virulence in mixed infections may simply lead to selection for greater virulence among coinfecting parasites. Similarly, hosts may invest less in their own defenses and may promote the growth of less virulent parasites that offer protection against more virulent parasites, thus accelerating the transition from parasitism to mutualism. However, the host will not promote the growth of a defensive parasite unless it provides a net benefit; in our model this is most likely when host protection occurs through resistance rather than tolerance due to ecological feedbacks that decrease (resistance) or increase (tolerance) the prevalence of another parasite (Fig. 1C). While the above scenarios seem plausible, the mathematical details will need to be worked out in future studies that account for coevolutionary interactions. Indeed, a greater theoretical understanding of mixed infections beyond the realm of virulence evolution is needed to support a growing body of empirical research, especially on microbe‐mediated protection in animal and plant hosts.

Associate Editor: A. Gardner

Handling Editor: J. Slate

## Supporting information


**Additional methods and results**.

**Source code (C++) for the simulations**.
