# Supplementary material for: Friendly foes: The evolution of host protection by a parasite
Source: Evol Lett. 2017 Aug 31;1(4):211–21. doi: 10.1002/evl3.19 (PMC6121858; doi:10.1002/evl3.19)
Supplement: Supplementary file 1 — Additional methods and results. [file EVL3-1-211-s001.pdf]

Friendly foes: the evolution of host protection by a parasite (supplementary material)

Ben Ashby[a,b,*] and Kayla C King[c]

a. Department of Mathematical Sciences, University of Bath, Bath, BA2 7AY, UK

b. Integrative Biology, University of California Berkeley, Berkeley, CA, USA

c. Department of Zoology, University of Oxford, Oxford, OX1 3PS, UK

* Corresponding author: benashbyevo@gmail.com

S1. Model A

S1.1 Derivation of $\mathrm{R}_0(\boldsymbol{i},\boldsymbol{j})$

In the main text we define $\mathrm{R}_0(i,j)$ to be the average number of secondary infections for parasite j when rare given that parasite i is already at equilibrium (Choisy and de Roode 2010). Following Choisy & de Roode (2010) we assume that $I_{12} \ll I_i^*$ so that the Jacobians for parasites 1 and 2 when rare are:

$$J_{21} = \begin{pmatrix} \beta_1(y)S^* - \Gamma_1(y) - \beta_2(y)I_2^* & \beta_1(y)S^* + \gamma_2 \\ I_2^*(\beta_1(y) + \beta_2(y)) & \beta_1(y)I_2^* - \Gamma_{12}(y) \end{pmatrix} \tag{S1a}$$

$$J_{12} = \begin{pmatrix} \tilde{\beta}_2 S^* - \Gamma_2 - \beta_1(y)I_1^* & \tilde{\beta}_2 S^* + \gamma_1 \\ I_1^*(\beta_1(y) + \beta_2(y)) & \beta_2(y)I_1^* - \Gamma_{12}(y) \end{pmatrix} \tag{S1b}$$

We now split the Jacobians into components F_{ij} and $V_{ij} = F_{ij} - J_{ij}$ such that:

$$F_{12} = \beta_1(y)\begin{pmatrix} S^* & S^* \\ I_2^* & I_2^* \end{pmatrix} \tag{S2a}$$

$$F_{21} = \begin{pmatrix} \tilde{\beta}_2 S^* & \tilde{\beta}_2 S^* \\ \beta_2(y)I_1^* & \beta_2(y)I_1^* \end{pmatrix} \tag{S2b}$$

$$V_{12} = \begin{pmatrix} \Gamma_1(y) + \beta_2(y)I_2^* & -\gamma_2 \\ -\beta_2(y)I_2^* & \Gamma_{12}(y) \end{pmatrix} \tag{S2c}$$

$$V_{12} = \begin{pmatrix} \Gamma_2 + \beta_1(y)I_1^* & -\gamma_1 \\ -\beta_1(y)I_1^* & \Gamma_{12}(y) \end{pmatrix} \tag{S2d}$$

$\mathrm{R}_0(i,j)$ is then equal to the dominant eigenvalue of the matrix $F_{ij}V_{ij}^{-1}$, as shown in equation 4 in the main text.

S1.2 Derivation of parasite fitness

When the resident is at equilibrium ($N^* = S^* + I_1^* + I_2^* + I_{12}^*$) the dynamics of a rare mutant are given by:

$$\frac{dI_m}{dt} = \lambda_1(y_m)S^* - [\Gamma_1(y_m) + \lambda_2^*(y_m)]I_m + \gamma_2 I_{m2} \tag{S3a}$$

$$\frac{dI_{m2}}{dt} = \lambda_1(y_m)I_2^* + \lambda_2^*(y_m)I_m - \Gamma_{12}(y_m)I_{m2} \tag{S3b}$$

To derive an expression for the invasion fitness of a rare mutant, we first calculate the Jacobian of these dynamics:

$$J = \begin{pmatrix} \beta_1(y_m)S^* - \Gamma_1(y_m) - \lambda_2^*(y_m) & \beta_1(y_m)S^* + \gamma_2 \\ \beta_1(y_m)I_2^* + \lambda_2^*(y_m) & \beta_1(y_m)I_2^* - \Gamma_{12}(y_m) \end{pmatrix} \tag{S4}$$

Next, we split the Jacobian into components F and V such that $V = F - J$:

$$F = \beta_1(y_m)\begin{pmatrix} S^* & S^* \\ I_2^* & I_2^* \end{pmatrix} \tag{S5a}$$

$$V = \begin{pmatrix} \Gamma_1(y_m) + \lambda_2^*(y_m) & -\gamma_2 \\ -\lambda_2^*(y_m) & \Gamma_{12}(y_m) \end{pmatrix} \tag{S5b}$$

The next-generation matrix, N_G, is then given by $N_G = FV^{-1}$, and the fitness of the mutant is sign equivalent to the dominant eigenvalue of N_G minus 1 (Hurford et al. 2010):

$$w(y_m) = \frac{\beta_1(y_m)\{S^*[\Gamma_{12}(y_m) + \lambda_2^*(y_m)] + I_2^*[\Gamma_1(y_m) + \gamma_2 + \lambda_2^*(y_m)]\}}{\Gamma_{12}(y_m)[\Gamma_1(y_m) + \lambda_2^*(y_m)] - \gamma_2\lambda_2^*(y_m)} - 1 \tag{S6}$$

S1.3 Additional results (virulence costs)

In the main text we present results for the case where there is a transmission rate cost associated with host protection. Here, we present additional results for the case where host protection leads to a cost in terms of virulence such that $\alpha_1(y) = \tilde{\alpha}_1(1 + c(y))$ (Figs. S1-S2). The relationship between the nature of the trade-off (i.e. its shape and strength) and the qualitative evolutionary outcome is broadly similar to the results described in the main text for a transmission rate cost (Figs. 2, 4). This is particularly evident for larger baseline levels of virulence for the protective parasite (compare Fig. 2C-D with Fig. S1C-D and Fig. 4C-D with Fig. S2C-D). The only notable exception is that we did not find repellers in isolation when the baseline level of virulence is low and the parasite confers resistance to the host (compare Fig. 2A-B with Fig. S1A-B).

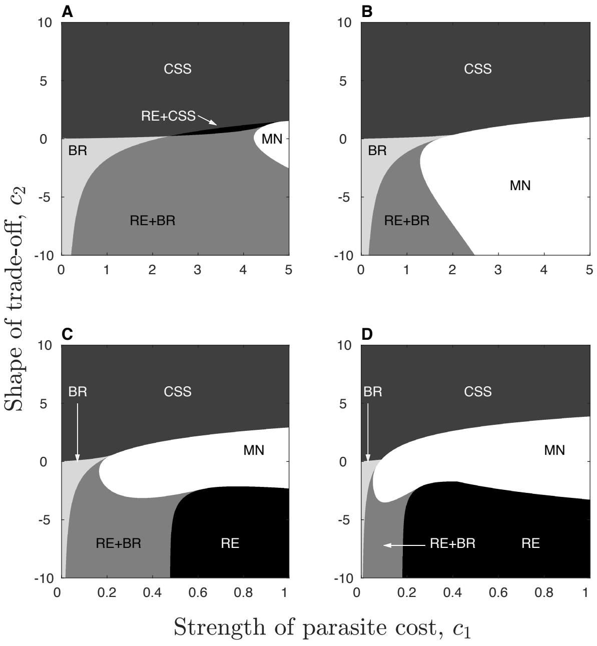

Figure S1 – Evolution of parasite-conferred resistance when host protection is associated with a cost in terms of virulence. Higher values of c_1 correspond to greater costs of defending against parasite 2, and higher (lower) values of c_2 correspond to more strongly accelerating (decelerating) costs (equation 1). The singular strategies are: minimisation (MN); intermediate continuously stable strategy (CSS); repeller (RE); and evolutionary branching (BR). The natural mortality rate, b, increases from 0.05 in the plots on the left to 0.5 on the right. The baseline virulence of parasite 1, $\tilde{\alpha}_1$, increases from 0.1 in the top row to 1 in the bottom row. Remaining parameters as described in Fig. 1 in the main text.

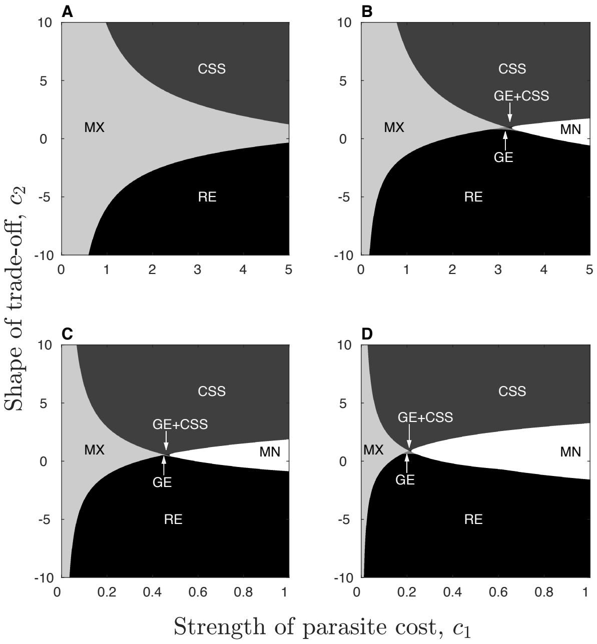

Figure S2 – Evolution of parasite-conferred tolerance when host protection is associated with a cost in terms of virulence. In addition to most of the singular strategies described in Fig. 2 for parasite-conferred resistance, we also find: maximisation (MX) for non-zero costs and Garden of Eden (GE) with or without a CSS. The natural mortality rate, b, increases from 0.05 in the plots on the left to 0.5 on the right. The baseline virulence of parasite 1, $\tilde{\alpha}_1$, increases from 0.1 in the top row to 1 in the bottom row. The cost function and the remaining labels and parameters are as described in Fig. 2 in the main text.

S2. Model B

S2.1 Model description

In the main text we explore a model of coinfection between different parasite species (model A). The model assumes that if a mutant strain arises in a given host it is either immediately cleared or it rapidly replaces the resident strain, which allows us to derive a relatively straightforward expression for the invasion fitness of the mutant. Here, we relax this assumption so that coinfections can also occur between the resident and mutant strains of parasite 1. However, this means we must also track double infections by the resident strain (I_{11}, I_{112}), otherwise an initially rare mutant has a frequency dependent advantage over the resident (Alizon 2013). We assume that hosts infected with strains i and j of parasite 1 experience susceptibility to parasite 2 equal to $[\lambda_2(y_i) + \lambda_2(y_j)]/2$ and I_{112} hosts have an infectious period equal to $2/[\Gamma_{12}(y_i) + \Gamma_{12}(y_j)]$. We assume there is no recovery from infection by either parasite ($\gamma_1 = \gamma_2 = 0$), as this greatly simplifies the derivation of parasite fitness. As with model A, we assume host protection is associated with a cost in terms of either the transmission rate, $\beta_1(y) = \tilde{\beta}_1(1 - c(y))$, or the virulence, $\alpha_1(y) = \tilde{\alpha}_1(1 + c(y))$, of parasite 1 (where $c(y)$ is defined in equation 1 in the main text).

The resident dynamics of model B are described by combining equation 2 in the main text (with $\gamma_1 = \gamma_2 = 0$) with equation S7 (and allowing for virulence costs such that $\Gamma_1(y) = \alpha_1(y) + b + \gamma_1$ and $\Gamma_{12}(y) = \Gamma_1(y) + \alpha_2(y) + \gamma_2$):

$$\frac{dI_{11}}{dt} = \lambda_1(y)I_1 - [\Gamma_1(y) + \lambda_2(y)]I_{11} \quad \text{(S7a)}$$

$$\frac{dI_{112}}{dt} = \lambda_1(y)I_{12} + \lambda_2(y)I_{11} - \Gamma_{12}(y)I_{12} \quad \text{(S7b)}$$

When the resident is at equilibrium ($N^* = S^* + I_1^* + I_2^* + I_{12}^* + I_{11}^* + I_{112}^*$), the initial dynamics of a rare mutant are:

$$\frac{dI_m}{dt} = \lambda_1(y_m)S^* - [\Gamma_1(y_m) + \lambda_1^*(y) + \lambda_2^*(y_m)]I_m \quad \text{(S8a)}$$

$$\frac{dI_{m1}}{dt} = \lambda_1(y_m)I_1^* + \lambda_1^*(y)I_m - \left(\Gamma_1(y_m) + \frac{1}{2}[\lambda_2^*(y) + \lambda_2^*(y_m)]\right)I_{m1} \quad \text{(S8b)}$$

$$\frac{dI_{m2}}{dt} = \lambda_1(y_m)I_2^* + \lambda_2^*(y_m)I_m - \left(\Gamma_{12}(y_m) + \lambda_1^*(y)\right)I_{m2} \quad \text{(S8c)}$$

$$\frac{dI_{m12}}{dt} = \lambda_1(y_m)I_{12}^* + \lambda_1^*(y)I_{m2} + \frac{1}{2}[\lambda_2^*(y) + \lambda_2^*(y_m)]I_{m1} - \frac{1}{2}\left(\Gamma_{12}(y) + \Gamma_{12}(y_m)\right)I_{m12} \quad \text{(S8d)}$$

where I_m and I_{m2} are as described for model A in the main text, I_{m1} is hosts coinfected with the mutant and resident strains of parasite 1, and I_{m12} is hosts additionally coinfected with parasite 2. The remaining aspects of model B and its analysis are identical to those for model A.

S2.2 Results

To derive an expression for invasion fitness in model B, we first calculate the Jacobian of the mutant dynamics (equation S8):

$$J = \begin{pmatrix} \beta_1(y_m)S^* - \Gamma_1(y_m) - \lambda_1^*(y) - \lambda_2^*(y_m) & \beta_1(y_m)S^* & \beta_1(y_m)S^* & \beta_1(y_m)S^* \\ \beta_1(y_m)I_1^* + \lambda_1^*(y) & \beta_1(y_m)I_1^* - \Gamma_1(y_m) - \frac{1}{2}[\lambda_2^*(\mathrm{y}) + \lambda_2^*(y_m)] & \beta_1(y_m)I_1^* & \beta_1(y_m)I_1^* \\ \beta_1(y_m)I_2^* + \lambda_2^*(y_m) & \beta_1(y_m)I_2^* & \beta_1(y_m)I_2^* - \Gamma_{12}(y_m) - \lambda_1^*(y) & \beta_1(y_m)I_2^* \\ \beta_1(y_m)I_{12}^* & \beta_1(y_m)I_{12}^* + \frac{1}{2}[\lambda_2^*(\mathrm{y}) + \lambda_2^*(y_m)] & \beta_1(y_m)I_{12}^* + \lambda_1^*(y) & \beta_1(y_m)I_{12}^* - \frac{1}{2}\left(\Gamma_{12}(\mathrm{y}) + \Gamma_{12}(y_m)\right) \end{pmatrix} \quad \text{(S9)}$$

Next, we split the Jacobian into components F and V such that $V = F - J$:

$$F = \beta_1(y_m)\begin{pmatrix} S^* & S^* & S^* & S^* \\ I_1^* & I_1^* & I_1^* & I_1^* \\ I_2^* & I_2^* & I_2^* & I_2^* \\ I_{12}^* & I_{12}^* & I_{12}^* & I_{12}^* \end{pmatrix} \quad \text{(S10a)}$$

$$V = \begin{pmatrix} \Gamma_1(y_m) + \lambda_1^*(y) + \lambda_2^*(y_m) & 0 & 0 & 0 \\ -\lambda_1^*(y) & \Gamma_1(y_m) + \frac{1}{2}[\lambda_2^*(\mathrm{y}) + \lambda_2^*(y_m)] & 0 & 0 \\ -\lambda_2^*(y_m) & 0 & \Gamma_{12}(y_m) + \lambda_1^*(y) & 0 \\ 0 & -\frac{1}{2}[\lambda_2^*(\mathrm{y}) + \lambda_2^*(y_m)] & -\lambda_1^*(y) & \frac{1}{2}\left(\Gamma_{12}(\mathrm{y}) + \Gamma_{12}(y_m)\right) \end{pmatrix} \quad \text{(S10b)}$$

The next-generation matrix, N_G, is equal to the product of F and the inverse of V:

$$N_G = FV^{-1} = \begin{pmatrix} S^* \\ I_1^* \\ I_2^* \\ I_{12}^* \end{pmatrix} \otimes \begin{pmatrix} C(y_m) \\ D(y_m) \\ E(y_m) \\ F(y_m) \end{pmatrix} \tag{S11}$$

where ⊗ is the outer matrix product and

$$C(y_m) = \frac{\beta_1(y_m)}{\Gamma_1(y_m) + \lambda_1^*(y) + \lambda_2^*(y_m)} \left(1 + \frac{\lambda_1^*(y)}{\Gamma_1(y_m) + \frac{1}{2}[\lambda_2^*(y) + \lambda_2^*(y_m)]} + \frac{\lambda_2^*(y_m)}{\Gamma_{12}(y_m) + \lambda_1^*(y)} \right.$$
$$\left. + 2\frac{\lambda_1^*(y)\left\{\Gamma_1(y_m)\lambda_2^*(y_m) + \frac{1}{2}[\lambda_2^*(y) + \lambda_2^*(y_m)][\Gamma_{12}(y_m) + \lambda_1^*(y) + \lambda_2^*(y_m)]\right\}}{\left\{\Gamma_1(y_m) + \frac{1}{2}[\lambda_2^*(y) + \lambda_2^*(y_m)]\right\}\{\Gamma_{12}(y_m) + \lambda_1^*(y)\}\{\Gamma_{12}(y) + \Gamma_{12}(y_m)\}} \right) \tag{S12a}$$

$$D(y_m) = \frac{\beta_1(y_m)}{\Gamma_1(y_m) + \frac{1}{2}[\lambda_2^*(y) + \lambda_2^*(y_m)]}\left\{1 + \frac{\lambda_2^*(y) + \lambda_2^*(y_m)}{\Gamma_{12}(y) + \Gamma_{12}(y_m)}\right\} \tag{S12b}$$

$$E(y_m) = \frac{\beta_1(y_m)}{\Gamma_{12}(y_m) + \lambda_1^*(y)}\left\{1 + \frac{2\lambda_1(y)}{\Gamma_{12}(y) + \Gamma_{12}(y_m)}\right\} \tag{S12c}$$

$$F(y_m) = \frac{2\beta_1(y_m)}{\Gamma_{12}(y) + \Gamma_{12}(y_m)} \tag{S12d}$$

The invasion fitness of the mutant is then sign equivalent to the dominant eigenvalue of N_G minus 1 (Hurford et al. 2010):

$$w(y_m) = S^*C(y_m) + I_1^*D(y_m) + I_2^*E(y_m) + I_{12}^*F(y_m) - 1 \tag{S13}$$

The selection gradient, $s(y) = \left.\frac{dw}{dy_m}\right|_{y_m=y}$, is:

$$s(y) = S^*\left.\frac{dC}{dy_m}\right|_{y_m=y} + I_1^*\left.\frac{dD}{dy_m}\right|_{y_m=y} + I_2^*\left.\frac{dE}{dy_m}\right|_{y_m=y} + I_{12}^*\left.\frac{dF}{dy_m}\right|_{y_m=y} \tag{S14}$$

As was the case with model A, we solve the ecological and evolutionary dynamics of the system numerically since there is no analytic solution for the equilibrium of the system. We compare the evolution of resistance and tolerance in models A and B (with no recovery in either model) when there is a transmission rate cost (Fig. S3) and when there is a virulence cost (Fig. S4). The differences between the two models are minimal in both cases. For example, branching of parasite-conferred resistance is slightly less likely in model B than in model A, and minimisation (maximisation) of tolerance is slightly more (less) likely in model B. These patterns hold regardless of whether costs affect the transmission rate or virulence. Hence, it seems that neither recovery nor coinfections between different strains of parasite 1 appear to have a significant impact on the qualitative results of the model.

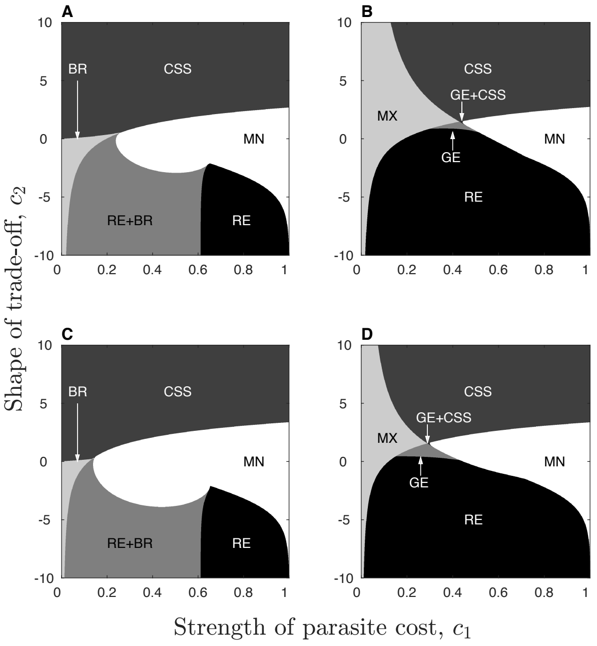

Figure S3 – Comparison of model A (no coinfection among different strains of parasite 1; top row) and model B (coinfection among different strains of parasite 1; bottom row) when there is a transmission rate cost, with no recovery from infection in either model ($\gamma_1 = \gamma_2 = 0$). The left (right) column shows the evolution of resistance (tolerance). The cost function, labelling, and remaining parameters are as described in Fig. 2 and 4, except $b = 0.5$ and $\tilde{\alpha}_1 = 0.1$.

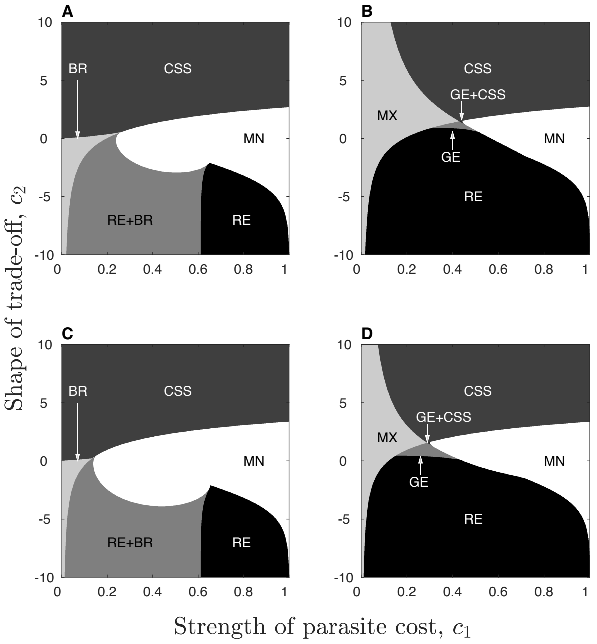

Figure S4 – Comparison of model A (no coinfection among different strains of parasite 1; top row) and model B (coinfection among different strains of parasite 1; bottom row) when there is a virulence cost, with no recovery from infection in either model ($\gamma_1 = \gamma_2 = 0$). The left (right) column shows the evolution of resistance (tolerance). The cost function, labelling, and remaining parameters are as described in Fig. 2 and 4, except $b = 0.5$ and $\tilde{\alpha}_1 = 0.1$.

References

Alizon, S. 2013. Co-infection and super-infection models in evolutionary epidemiology. Interface Focus 3:20130031.

Choisy, M., and J. C. de Roode. 2010. Mixed infections and the evolution of virulence: effects of resource competition, parasite plasticity, and impaired host immunity. Am. Nat. 175:E105-18.

Hurford, A., D. Cownden, and T. Day. 2010. Next-generation tools for evolutionary invasion analyses. J. R. Soc. Interface 7:561–571.
